# *In vitro* efficacy of sulbactam/durlobactam combined with β-lactam antibiotics in Australian *Mycobacterium abscessus* isolates

**DOI:** 10.1093/jac/dkaf441

**Published:** 2025-12-12

**Authors:** Kirby Patterson-Fahy, Robyn Carter, Scott C Bell, Ieuan E S Evans, Andrew John Burke, Rachel M Thomson

**Affiliations:** The University of Queensland, Greenslopes Clinical School, Gallipoli Medical Research, Greenslopes, QLD, Australia; The University of Queensland, Greenslopes Clinical School, Gallipoli Medical Research, Greenslopes, QLD, Australia; Department of Thoracic Medicine, The Prince Charles Hospital, Chermside, Brisbane, QLD, Australia; Child Health Research Centre, The University of Queensland, South Brisbane, QLD, Australia; Department of Respiratory Medicine, Gold Coast University Hospital, Southport, QLD, Australia; School of Medicine and Dentistry, Health Group, Griffith University, Southport, QLD, Australia; Department of Thoracic Medicine, The Prince Charles Hospital, Chermside, Brisbane, QLD, Australia; Faculty of Medicine, The University of Queensland, Mayne Medical School, Herston, Brisbane, Australia; Faculty of Medicine, The University of Queensland, Mayne Medical School, Herston, Brisbane, Australia; Department of Infectious Disease, The Prince Charles Hospital, Chermside, Brisbane, QLD, Australia; The University of Queensland, Greenslopes Clinical School, Gallipoli Medical Research, Greenslopes, QLD, Australia; Department of Thoracic Medicine, The Prince Charles Hospital, Chermside, Brisbane, QLD, Australia; MetroSouth Clinical TB Service, Princess Alexandra Hospital, Brisbane, Australia

## Abstract

**Background and objectives:**

*Mycobacterium abscessus* has extensive innate and acquired antibiotic resistance resulting in limited antibiotic treatment options and poor clinical outcomes. Currently, the only β-lactam antibiotics with efficacy against *M. abscessus* are imipenem and cefoxitin. Durlobactam is a β-lactamase inhibitor that may overcome intrinsic resistance mechanisms and enable the use of alternative oral β-lactam antibiotics. The objective of this study was to determine whether sulbactam/durlobactam increases the susceptibility of *M. abscessus* to alternative β-lactam antibiotics.

**Material and methods:**

Antibiotic susceptibility testing was performed for durlobactam, meropenem, cefuroxime/amoxicillin alone, and sulbactam/durlobactam alone and in combination with meropenem and cefuroxime/amoxicillin according to Clinical Laboratory Standards Institute (CLSI) standards. These results were then compared with imipenem susceptibility with and without relebactam.

**Results:**

Sulbactam/durlobactam significantly lowered the MICs of *M. abscessus* to meropenem, cefuroxime and cefuroxime/amoxicillin to MICs comparable to those of imipenem and imipenem/relebactam. The culture medium used had a significant impact on MIC, with Middlebrook 7H9 having significantly lower MICs for all combinations containing durlobactam compared with CLSI standard CAMHB media.

**Conclusion:**

Sulbactam/durlobactam significantly increased susceptibility to oral and intravenous β-lactam antibiotics in the form of cefuroxime, cefuroxime/amoxicillin and meropenem against clinical isolates of *M. abscessus*. This study also found significant differences in susceptibility to β-lactam antibiotics dependent on the culture media used, highlighting that the optimal culture methods for determining MIC in *M. abscessus* remains uncertain. Future *in vivo* studies are required to determine whether the *in vitro* efficacy of the β-lactam combinations studied could result in clinical efficacy for *M. abscessus* disease.

## Introduction

The prevalence of non-tuberculous mycobacterial pulmonary disease has been increasing globally, particularly in Queensland, Australia.^1^  *Mycobacterium abscessus* (Mabs) has emerged as a major opportunistic pathogen causing non-tuberculous mycobacterial pulmonary disease with significant associated morbidity and mortality.^1,2^ Treatment options for Mabs remain limited with the optimal treatment remaining unknown. Successful treatment outcomes are estimated to be as low as 45% due to extensive innate and acquired antibiotic resistance as well as high toxicity and poor tolerability of currently available antibiotic therapies.^3,4^ Novel treatment options and strategies are therefore urgently required to improve the treatment outcomes of people with Mabs pulmonary disease.

Current clinical treatment guidelines recommend combination intravenous and oral antibiotics with an initial induction treatment phase followed by a continuation phase frequently up to 12–18 months duration.^5–7^ The β-lactam antibiotics imipenem and cefoxitin are recommended in the induction treatment phase where the use of imipenem has been correlated with treatment success.^3,4^ The activity of β-lactams was historically thought to rely on their action against penicillin binding proteins (PBPs) that catalyse the final step of peptidoglycan synthesis to cross link peptide side chains in bacteria.^8^ Mabs has most cross linkages catalysed by L,D-transpeptidases rather than PBPs^9^ with several classical PBPs and the evolutionarily distinct L,D-transpeptidases resulting in significant redundancy in the peptidoglycan synthesis pathway.^10^ Imipenem has been found to inhibit most of both PBPs^11^ and L,D-transpeptidases,^12^ which is thought to explain its efficacy against Mabs.

It is proposed that dual β-lactam therapy with non-redundant targets could significantly increase treatment efficacy.^13^  *In vitro* analysis has shown four patterns of β-lactam binding to PBPs in Mabs driven by antibiotic class, specifically: carbapenems, cephalosporins, β-lactamase inhibitors (BLI) and penicillins.^11^ This suggests combination β-lactam treatment strategies could impart greater efficacy, which has been demonstrated in *in vitro* testing in previous experimental work.^14–16^

Alongside interest in dual combination β-lactam therapy, there has been increasing focus on the use of BLI in the treatment of Mabs.^14,17^ Mabs harbours a single class A β-lactamase (Bla_MAB_) that is chromosomally encoded and conserved across subspecies.^18^ Bla_MAB_ hydrolysis of imipenem is slow, which is thought to contribute to imipenem's effectiveness compared with other β-lactams.^19^ In addition, *in vitro* testing has suggested that the addition of BLI to imipenem can be associated with improved efficacy against Mabs.^20–22^

Durlobactam, a novel diazabicyclooctane BLI is of increasing interest due to strong *in vitro* data suggesting it improves efficacy of companion β-lactam antibiotics against Mabs.^23–26^ Durlobactam is also reported to have direct activity against Mabs when used as a single drug.^24^  *In vitro* testing of durlobactam action against Mabs has shown that durlobactam binds to Bla_MAB_, two L,D-transpeptidases and Mabs D,D-carboxypeptidase supporting its potential mechanism of action.^24^ Durlobactam has recently gained FDA approval for the treatment of *Acinetobacter baumannii* complex pneumonia in the formulation of sulbactam/durlobactam. Sulbactam/durlobactam has also been shown to significantly reduce the MIC of both imipenem and cefoxitin.^25,26^

We aimed to perform antibiotic susceptibility testing on a diverse sample of 50 well-characterized Mabs isolates derived from pulmonary infections in Australia to explore the following: durlobactam susceptibility of Mabs, the impact of sulbactam on susceptibility to durlobactam, the effect of sulbactam/durlobactam on meropenem susceptibility as a potential alternative to imipenem and to explore the utility of sulbactam/durlobactam as a synergistic agent with oral β-lactams: cefuroxime and amoxicillin.

## Material and methods

### *M. abscessus* isolates

Fifty Mabs respiratory isolates underwent susceptibility testing, 25 isolated from patients with cystic fibrosis and 25 from patients without cystic fibrosis. The isolates used in this study formed a subset of those used to previously test a range of antibiotics including amikacin, clarithromycin and imipenem/relebactam.^22^ All isolates have undergone whole-genome sequencing, and added to the phylogeny of worldwide dominant circulating clones (DCC) isolates to identify their inter-relatedness enabling selection of a diverse representative sample of Mabs isolates. The isolate characteristics are summarized in Table 1. Isolates were classified as either rough or smooth based on the morphology of growth on Mueller–Hinton (MH) + 10% oleic acid albumin dextrose and catalase (OADC) agar incubated at 30°C.

**Table 1.** Characteristics of *M. abscessus* isolates

	Total	CF	Non-CF
*Mycobacterium abscessus*	50	25	25
subsp. *abscessus*	36	19	17
subsp. *massiliense*	14	6	8
Antibiotic resistance^a^			
Amikacin resistance	4	4	0
Constitutive clarithromycin resistance	1	1	0
Inducible clarithromycin resistance	27	17	10
Imipenem resistance	11	7	4
DCC^b^ type			
DCC1	12	7	5
DCC2	3	2	1
DCC3	5	1	4
DCC4	3	2	1
DCC5	8	4	4
DCC6	2	2	0
DCC7	1	0	1
non-DCC	16	7	9
Smooth	40	19	21
Rough	10	6	4

^a^Determined phenotypically according to CLSI criteria.^27^

^b^Dominant circulating clones.

Sequence data have been deposited in the European Nucleotide Archive (https://www.ebi.ac.uk/ena/browser/home) under accession ERP001039, and the NIH Sequence Read Archive (https://www.ncbi.nlm.nih.gov/sra/docs) under accession numbers PRJNA941035 and PRJEB2779.

### Antibiotics

Amoxicillin, cefuroxime and meropenem were sourced from Sigma-Aldrich. Sulbactam and durlobactam were sourced through Focus Biosciences. Meropenem and amoxicillin stock solutions were prepared in DMSO. Cefuroxime, sulbactam and durlobactam stock solutions were prepared in autoclaved milli-Q water. Stock solutions were stored at −80°C.

### Antibiotic susceptibility testing

Antibiotic susceptibility testing (AST) was performed by broth microdilution as per the CLSI guidelines.^28^ Mabs isolates were grown for 3 days on MH + 10% OADC agar before being used to create a 0.5 McFarland suspension. The 0.5 McFarland standard was used to create a suspension in CAMHB with TES broth media, which was then inoculated into a 96-well plate to have a final organism concentration of 5 × 10^5^ cfu/mL in each well. The 96-well plates were prepared with a 2-fold dilution of antibiotics in CAMHB media between 0.06 and 128 mg/L for amoxicillin and meropenem, and 0.06–64 mg/L for durlobactam in technical duplicate. *M. peregrinum* ATCC 700686 was run as the quality control strain according to CLSI guidelines.^28^ Durlobactam was tested alone and in a ratio of 1:1 with sulbactam. Sulbactam/durlobactam was added to other antibiotics at a fixed concentration of 4/4 µg/ml as per the FDA susceptible breakpoint for *Acinetobacter calcoaceticus–baumannii* complex.^29^ This concentration has been determined to be physiologically achievable^30^ and has previously been used in *in vitro* analysis targeting Mabs.^25^ Sulbactam/durlobactam was added to other antibiotics rather than durlobactam alone as that is the only form that is currently commercially available.^31^ Combination AST was conducted for cefuroxime/amoxicillin with additional AST for cefuroxime/amoxicillin in combination with sulbactam/durlobactam. Amoxicillin was added at a fixed concentration of 8 mg/L aligning with what has previously been used in *in vitro* experimental work targeting Mabs.^24,32^ MICs were read after 3 days of incubation at 30°C or 4 days of incubation for isolates that had insufficient growth at 3 days. Use of the CLSI method also enabled comparison of the MICs determined in this project with previous AST results for the same isolates.^22^

### Growth media comparison

Two Mabs clinical isolates, one subsp. *abscessus* DCC1 and one subsp. *massiliense* DCC3, and the laboratory strains *M. abscessus* ATCC 19977^T^ and *M. peregrinum* ATCC 700686^T^, underwent comparative AST in both CAMHB and 7H9 + 10% albumin dextrose and catalase (ADC) + 0.02% glycerol media. AST was performed as described before at 30°C incubation temperature, with antibiotic dilutions prepared in the appropriate media and a single 0.5 McFarland standard used to create inoculum suspensions in both media types.

### Statistical analysis

MIC values were log_2_ transformed and GraphPad Prism version 10.3.1 was used for all statistical analyses. One-way ANOVA followed by Tukey multiple comparisons was used to determine statistical significance of differences in MICs.

## Results

**Figure 1.**
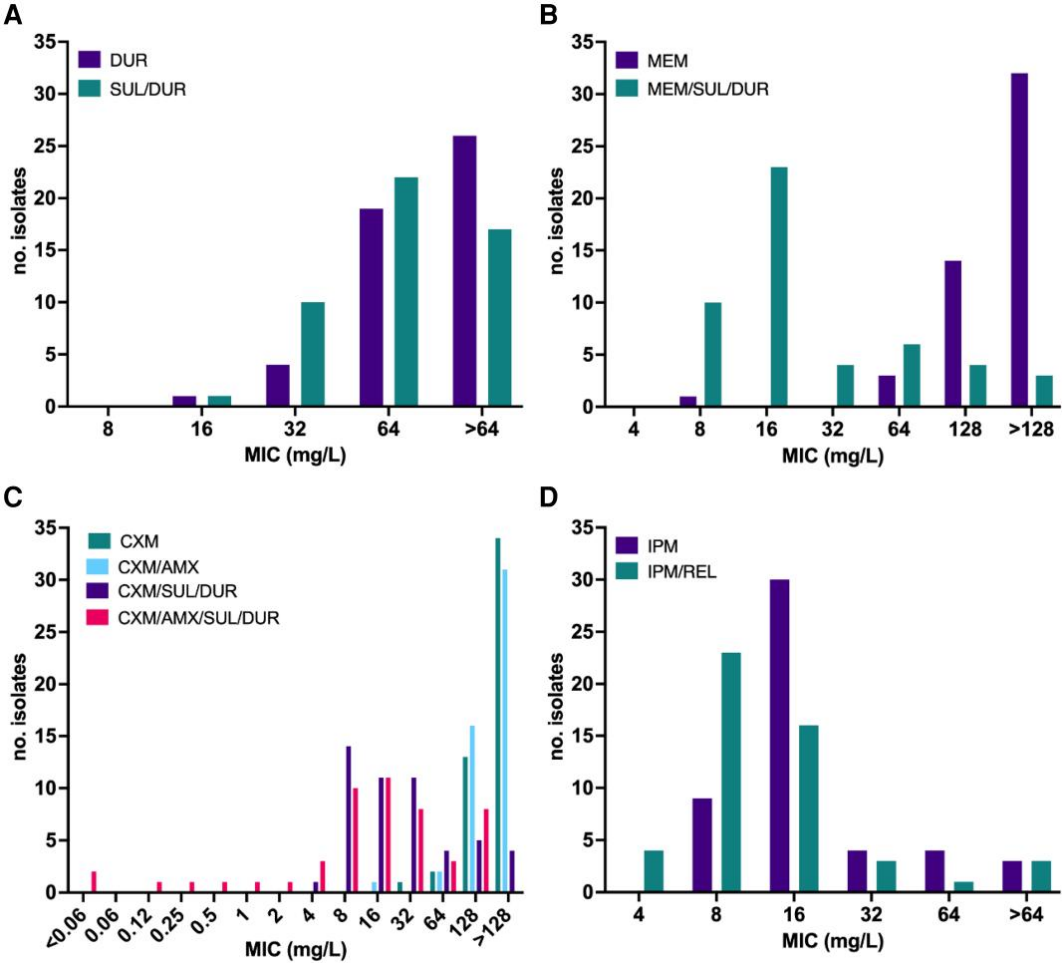
MIC distributions of 50 Australian *M. abscessus* isolates. (a) Durlobactam and sulbactam/durlobactam. (b) Meropenem and meropenem + sulbactam/durlobactam. (c) Cefuroxime, cefuroxime/amoxicillin, cefuroxime + sulbactam/durlobactam and cefuroxime/amoxicillin/sulbactam/durlobactam. (d) Imipenem and imipenem/relebactam. Amoxicillin added at a fixed concentration of 8 mg/L. Sulbactam/durlobactam added at a fixed concentration of 4/4 mg/L. Relebactam added at a fixed concentration of 4 mg/L. CXM, cefuroxime; AMX, amoxicillin; SUL/DUR, sulbactam/durlobactam; MEM, meropenem; IPM, imipenem; REL, relebactam.

Durlobactam exhibited limited inhibitory activity against the tested Mabs isolates with a MIC_50_ of >64 mg/L (Table 2, Figure 1a). The addition of sulbactam to durlobactam reduced the MIC_50_ to 64 mg/L approaching statistical significance (*P* = 0.051). The Mabs isolates tested were also highly resistant to cefuroxime with a MIC_50_ of >128 mg/L (Table 2, Figure 1c). The dual β-lactam combination cefuroxime/amoxicillin did not have a significantly lower MIC than cefuroxime alone [Figure 1c, Figure S1 (available as Supplementary data at *JAC* Online)]. The addition of sulbactam/durlobactam to cefuroxime significantly reduced the MIC_50_ by 8-fold to 16 mg/L. The combination of cefuroxime/amoxicillin/sulbactam/durlobactam (CXM/AMX/SUL/DUR) had no change in MIC_50_ compared with cefuroxime/sulbactam/durlobactam (CXM/SUL/DUR). However, the distribution of MICs was markedly different with a significant number of isolates exhibiting MICs of ≤4 mg/L (Figure 1c) with these isolates (10/50, 20%) now being classed as susceptible according to imipenem and meropenem CLSI interpretive criteria.^27^

**Table 2.** MIC_50_, MIC_90_ and range determined for 50 Australian Mabs isolates. The CLSI breakpoints for imipenem and meropenem are 8–16 mg/L is intermediate.^27^ Amoxicillin added at a fixed concentration of 8 mg/L. Sulbactam/durlobactam added at a fixed concentration of 4/4 mg/L. Relebactam added at a fixed concentration of 4 mg/L

	MIC (mg/L)		
Antibiotic	MIC50	MIC90	Range
IPM	16	64	8–>64
IPM/REL	8	32	4–>64
DUR	>64	>64	32–>64
SUL/DUR	64	>64	32–>64
MEM	>128	>128	64–>128
MEM/SUL/DUR	16	128	8–>128
CXM	>128	>128	64–>128
CXM/SUL/DUR	16	128	8–>128
CXM/AMX	>128	>128	64–128
CXM/AMX/SUL/DUR	16	128	<0.06–128

CXM, cefuroxime; AMX, amoxicillin; SUL/DUR, sulbactam/durlobactam; MEM, meropenem; IPM, imipenem; REL, relebactam.

**Figure 2.**
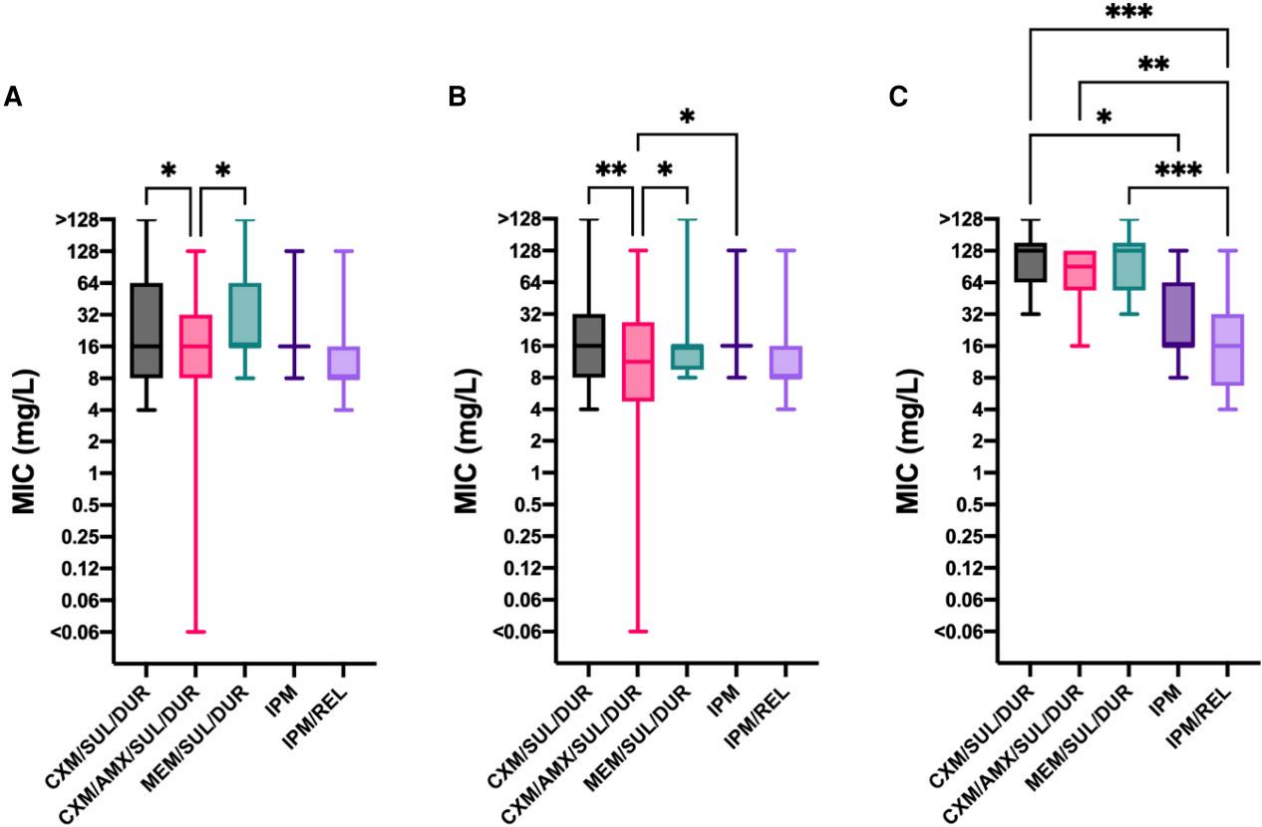
Imipenem and imipenem/relebactam susceptibility compared with alternative β-lactams with sulbactam/durlobactam. (a) All isolates (*n* = 50). (b) Smooth isolates (*n* = 40). (c) Rough isolates (*n* = 10). Amoxicillin added at a fixed concentration of 8 mg/L. Sulbactam/durlobactam added at a fixed concentration of 4/4 mg/L. Relebactam added at a fixed concentration of 4 mg/L. CXM, cefuroxime; AMX, amoxicillin; SUL/DUR, sulbactam/durlobactam; MEM, meropenem; IPM, imipenem; REL, relebactam. Only statistically significant associations are shown, all others are non-significant *P ≤ 0.05, **P ≤ 0.01, ***P ≤ 0.001.

The MIC_50_ for meropenem also demonstrated a high level of resistance compared with imipenem, >128 and 16 mg/L, respectively (Table 2). None of the Mabs clinical isolates tested were susceptible to meropenem in isolation, a single isolate was within the intermediate range and all others were resistant. However, the addition of sulbactam/durlobactam to meropenem resulted in a >8-fold reduction in MIC_50_ to 16 mg/L (Table 2, Figure 1b). The addition of sulbactam/durlobactam to cefuroxime, cefuroxime/amoxicillin and meropenem all resulted in MICs equivalent to those for imipenem and imipenem/relebactam (Figure 2). Imipenem resistant isolates did not have statistically significantly different MICs from meropenem/sulbactam/durlobactam (MEM/SUL/DUR), CXM/SUL/DUR or CXM/AMX/SUL/DUR compared with the imipenem susceptible isolates (Figure S2).

The morphotype of the isolates significantly affected the antibiotic susceptibility with smooth isolates having lower MIC_50_ (Figure 2b) than rough isolates (Figure 2c). Sulbactam/durlobactam did not significantly reduce the meropenem, cefuroxime or cefuroxime/amoxicillin MIC for the rough isolates (Figures S1c and S3c). However, for smooth isolates, the CXM/AMX/SUL/DUR MIC was significantly lower than the imipenem MIC (Figure 2b). CXM/AMX/SUL/DUR was equivalent to imipenem for the rough isolates, where imipenem/relebactam had a significantly lower MIC than all sulbactam/durlobactam combinations (Figure 2c).

**Figure 3.**
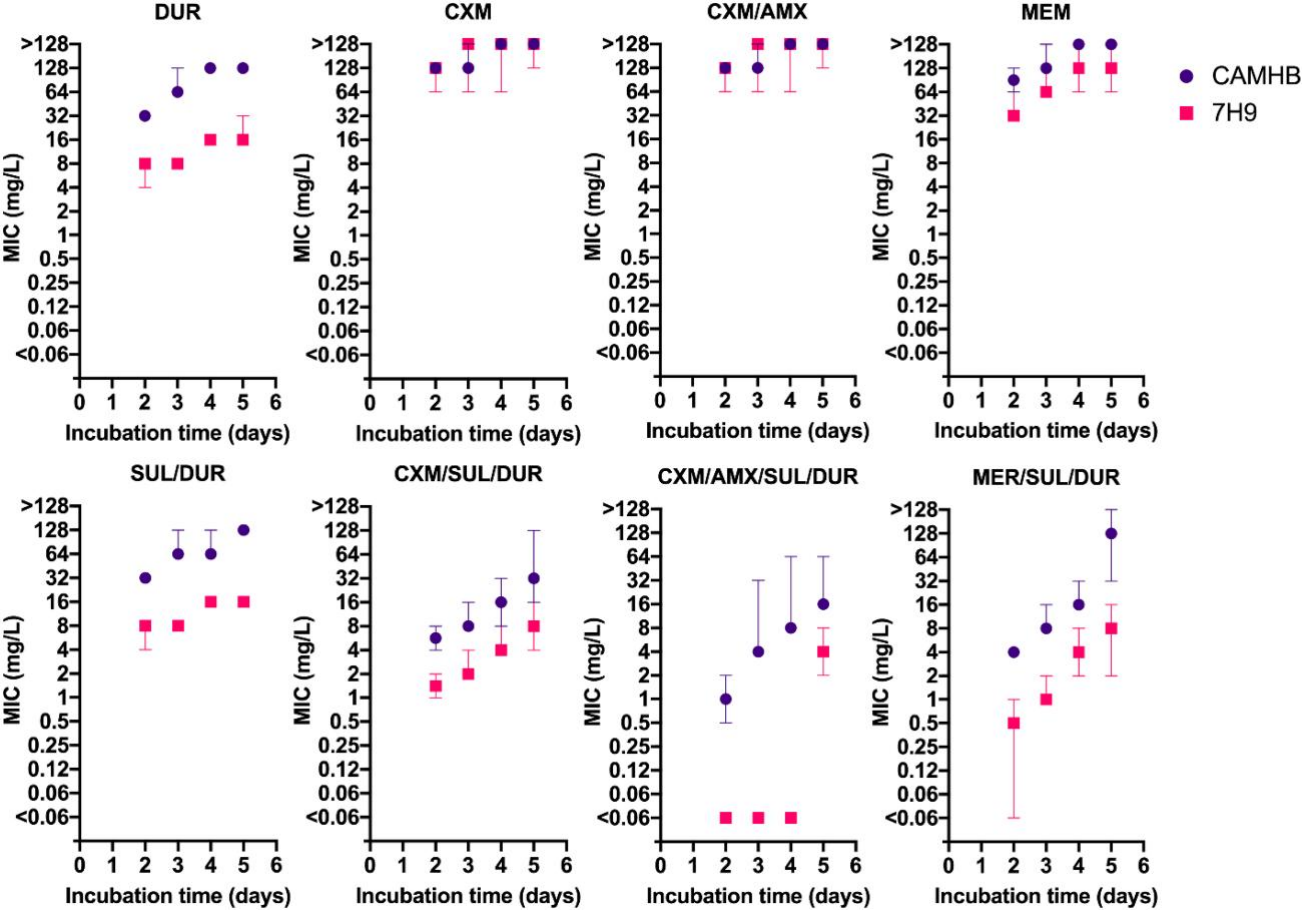
Antibiotic susceptibility using both CAMHB and Middlebrook 7H9 culture media. The MICs increased with increasing incubation period for all antibiotic combinations. MICs in Middlebrook 7H9 were lower than those in CAMHB. Three *M. abscessus* isolates including ATCC 19977^T^, a subsp. *abscessus* and a subsp. *massiliense* clinical isolate were tested. Data are represented as the median and range. Amoxicillin added at a fixed concentration of 8 mg/L. Sulbactam/durlobactam added at a fixed concentration of 4/4 mg/L. Relebactam added at a fixed concentration of 4 mg/L. CXM, cefuroxime; AMX, amoxicillin; SUL/DUR, sulbactam/durlobactam; MEM, meropenem; IPM, imipenem; REL, relebactam.

The reference strain, *M. abscessus* ATCC 19977^T^ and two clinical isolates, one subsp. *abscessus* and one subsp. *massiliense*, underwent comparative AST in both CAMHB and Middlebrook 7H9 media in parallel. MICs determined in CAMHB were greater than MICs determined in Middlebrook 7H9 media for durlobactam, sulbactam/durlobactam, meropenem and all other combinations containing sulbactam/durlobactam irrespective of incubation period (Figure 3).

## Discussion

Sulbactam/durlobactam significantly reduced the MIC of β-lactam antibiotics and combinations for Mabs isolated from people with and without cystic fibrosis. Our Mabs clinical isolates had high MICs (<128 mg/L) for cefuroxime, cefuroxime/amoxicillin and meropenem. However, the addition of sulbactam/durlobactam reduced the MIC_50_ of meropenem, cefuroxime and the combination of cefuroxime/amoxicillin to equivalence with imipenem and imipenem/relebactam. All of the MIC_50_ remained within the intermediate range of the CLSI breakpoint^27^ for carbapenems, however, *in vitro* susceptibility has not been correlated with treatment outcomes for imipenem^33^ despite imipenem use being correlated with treatment success.^3,4^

The MIC distributions for cefuroxime/amoxicillin were similar to cefuroxime alone, although interestingly the MICs were high (>128 mg/L) in contrast to previously published studies.^23^ The largest reduction in MICs was seen with the combination of CXM/AMX/SUL/DUR, with two isolates having MICs <0.06 mg/L. However, overall, the range of the MICs was wide, likely to be reflective of differing isolate morphology and the impact of this on antibiotic resistance. Given the MIC_50_ for MEM/SUL/DUR and CXM/AMX/SUL/DUR were equivalent to those of imipenem alone, it is possible that these combinations could represent viable alternatives to imipenem for treatment of Mabs disease. This is particularly important to consider in the context of poor antibiotic tolerance with guideline recommended antibiotic therapy.^3^ Furthermore, cefuroxime and amoxicillin can both be used as oral options potentially widening their application in the treatment of Mabs in the outpatient setting.

The MIC for durlobactam in isolation was high (>64 mg/L) compared with our clinical isolates. Although it did not reach statistical significance, the addition of sulbactam resulted in a 2-fold reduction in MIC consistent with previously published results for Mabs.^25^ While sulbactam does not inhibit Bla_MAB,_^19^ it has been found to bind to some PBPs.^11^ The reduction in MIC with sulbactam/durlobactam compared with durlobactam is unlikely to be clinically significant due to the MIC being greater than achievable plasma and intrapulmonary concentrations.^30^ The sulbactam/durlobactam MIC for Mabs also remains well above the resistance cut-off determined for the *A. baumanii* complex.^29^ It remains unknown whether sulbactam may contribute to the efficacy in combination with other beta-lactams. The high MICs for durlobactam and sulbactam/durlobactam contrasted with previous published results for Mabs having much lower MICs ∼8 and 4 mg/L^23–26^ compared with this study's >64 and 64 mg/L, respectively.

The MICs determined for the clinical isolates used in this study were significantly higher than recently published data on the same β-lactam combinations in Mabs (Table 3).^23,25,26^ There were no significant differences in susceptibility to antibiotics tested for isolates based on subspecies, DCC designation, whether the isolates were from people with CF or without CF, imipenem resistance, inducible clarithromycin resistanc, or amikacin resistance (Figures S2 and S4–S8). However, the morphotype of the isolates did impact on susceptibility. Smooth isolates had a significant (>8-fold) reduction in MIC with the addition of sulbactam/durlobactam compared with an only 2-fold reduction for rough isolates that was not statistically significant. Rough isolates have previously been found to be less susceptible to β-lactam and β-lactam-BLI combinations than smooth isolates^34^ and have been associated with worse treatment outcomes.^35^ However, the smooth isolates in this study still had significantly higher MICs than previous work, therefore these results cannot simply be explained by a larger proportion of rough isolates in this study. This suggests that a factor other than differences in isolate characteristics or origin was responsible for differing MIC results compared with previously published work. We propose this is probably related to the media used to conduct MIC experiments (Table 3).

**Table 3.** Susceptibility of *Mycobacterium abscessus* to sulbactam/durlobactam and β-lactams. The MIC_50_, MIC_90_ and range determined in this project as well as susceptibility testing method used compared with literature results

Study	No. isolates	Media	Temp (°C)	Incubation time (days)	DUR (mg/L)		SUL/DUR (mg/L)		CXM (mg/L)		CXM/SUL/DUR (mg/L)		CXM/AMX (mg/L)		CXM/AMX/SUL/DUR (mg/L)	
					MIC_50_	Range	MIC_50_	Range	MIC_50_	Range	MIC_50_	Range	MIC_50_	Range	MIC_50_	Range
Current study	50	CAMHB	30	3–4	>64	32–>64	64^a^	32–>64^a^	>128	64–>128	16	8–>128	>128	64–128	16	<0.06–128
Dousa *et al.*^24^	100	7H9	30	2	4	2–8			8	4–128	2^c^	0.5–4^c^	4	0.5–>64	≤0.06^c^	≤0.06–1^c^
Negatu *et al*.^25^	72	7H9	37	3	8	4–32	4^b^	2–32^b^								

AMX added at a fixed concentration of 8 mg/L. SUL/DUR added at a fixed concentration of 4/4 mg/L.

AMX, amoxicillin; CXM, cefuroxime; DUR, durlobactam; SUL, sulbactam; SUL/DUR, sulbactam/durlobactam; IPM, imipenem.

^a^SUL/DUR in a 1:1 ratio.

^b^SUL added at fixed concentration of 4 mg/L.

^c^DUR in isolation added at 1:1 ratio with CXM.

The CLSI broth microdilution method used in this study is the method recommended to be used by clinical guidelines.^5–7^ Previously published studies have used a modified method substituting the recommended CAMHB media for Middlebrook 7H9 media.^23–26^ β-lactams degrade at the incubation temperature recommended by the CLSI guidelines^36^ and the substitution of media has been thought to allow interpretation of the MIC in a shorter incubation period thus providing a more accurate MIC.^12,37^ To investigate the impact of incubation period and media, a subset of isolates including the Mabs ATCC strain 19977^T^ underwent AST in both CAMHB and Middlebrook 7H9 broth media simultaneously. The MIC results showed that regardless of incubation period the MIC ranges were different between the two media types for meropenem, durlobactam, sulbactam/durlobactam and all β-lactam combinations containing sulbactam/durlobactam. Mabs ATCC strain 19977^T^ had a 4-fold higher durlobactam MIC of 64 mg/L in CAMHB media compared with 8 mg/L in Middlebrook 7H9 after a standard 3-day incubation period. This is consistent with a recent study which reported the same elevated sulbactam/durlobactam MIC in CAMHB compared with 7H9 media.^38^ Comparison of MICs across the two media types with a larger number of isolates and range of antibiotics would further elucidate the potential effect of media on antibiotic susceptibility. CAMHB has a more alkaline pH than Middlebrook 7H9 media, which has been associated with greater β-lactam degradation^16^ and could be an alternative explanation to longer incubation period for the higher MICs in CAMHB.

The significant difference in MIC between media types shown in this study also brings into question whether the growth conditions *in vitro* are reflective enough of conditions *in vivo* to be predictive of clinical utility. Significant differences in MICs have also been reported for Mabs between Middlebrook 7H9 media and media designed to more closely resemble sputum^39^ as well as planktonic Mabs compared with Mabs in biofilm^40,41^ and macrophages.^40^  *In vitro* susceptibility data for Mabs has not been well correlated with clinical outcomes^33^ despite treatment guideline recommendations that AST can be used to guide antibiotic selection.^5–7^ The extended incubation time required for Mabs as well as antibiotic instability is likely to increase the difficulty of correlating AST with clinical outcomes especially for β-lactams such as imipenem.^37^

The *in vitro* susceptibility results determined in this study show significant reductions in MIC of meropenem, cefuroxime and cefuroxime/amoxicillin when used in combination with sulbactam/durlobactam for Mabs pulmonary isolates from people with and without cystic fibrosis. These results contribute to previous literature highlighting sulbactam/durlobactam efficacy in β-lactam combinations and strengthen support for further investigation of sulbactam/durlobactam in Mabs. The reduction to equivalent MIC levels seen for imipenem and efficacy in Mabs isolated from people with cystic fibrosis are encouraging for potential application in clinical practice. However, there are limitations of our study that need to be considered. AMX/SUL/DUR susceptibility and impact of durlobactam alone were also not assessed. Results from previous work suggest that cefuroxime/amoxicillin have synergy when used together^42^ and that there is greater efficacy in the addition of sulbactam/durlobactam to cefuroxime/amoxicillin than either cefuroxime or amoxicillin alone.^24^ Currently, durlobactam is only commercially available in combination with sulbactam,^31^ which was the justification for assessing only the sulbactam/durlobactam combination in this study.

This study was an *in vitro* study and inherently does not directly predict *in vivo* or clinical efficacy. Pharmacodynamic and pharmacokinetic studies are required to determine whether the β-lactam combinations tested achieve adequate concentrations to inhibit Mabs at the site of infection without toxicity. Previous studies in *Acinetobacter* complex pneumonia support efficacy of sulbactam/durlobactam with good tolerability although with a lower MIC and not for the length of antibiotic treatment typically required for Mabs.^43^ Time–kill assays^38^ and hollow-fibre models^44^ are currently being used to further investigate sulbactam/durlobactam β-lactam combinations *in vitro*. There is no clear pre-clinical to clinical pathway for Mabs disease due to significant limitations of current animal models.^45^ Several case studies have been reported using dual β-lactam treatments successfully for Mabs disease,^46^ however, the effect of dual β-lactam treatments among the multi-drug combinations used is difficult to elucidate. We believe that the highest quality evidence for clinical efficacy of sulbactam/durlobactam and other novel treatments for Mabs disease is likely to come from direct translation of promising *in vitro* studies into comprehensive clinical trials such as FORMaT.^47^

### Conclusion

In conclusion, the results of this study support ongoing evaluation of sulbactam/durlobactam as a novel treatment option for Mabs disease. The combination of sulbactam/durlobactam with meropenem, cefuroxime and cefuroxime/amoxicillin had MICs equivalent to those of imipenem for 50 Australian Mabs isolates. Future research is required to determine the clinical efficacy of sulbactam/durlobactam and durlobactam alone, as well as the effect of AST method on correlation of *in vitro* MICs with *in vivo* outcomes for β-lactams.

## Supplementary Material

dkaf441_Supplementary_Data

## Supplementary data

Figures S1 to S8 are available as Supplementary data at *JAC* Online.
