# Supplementary material for: In vitro efficacy of sulbactam/durlobactam combined with β-lactam antibiotics in Australian Mycobacterium abscessus isolates
Source: J Antimicrob Chemother. 2025 Dec 12;81(1):dkaf441. doi: 10.1093/jac/dkaf441 (PMC12802925; doi:10.1093/jac/dkaf441)
Supplement: dkaf441_Supplementary_Data [file dkaf441_supplementary_data.docx]

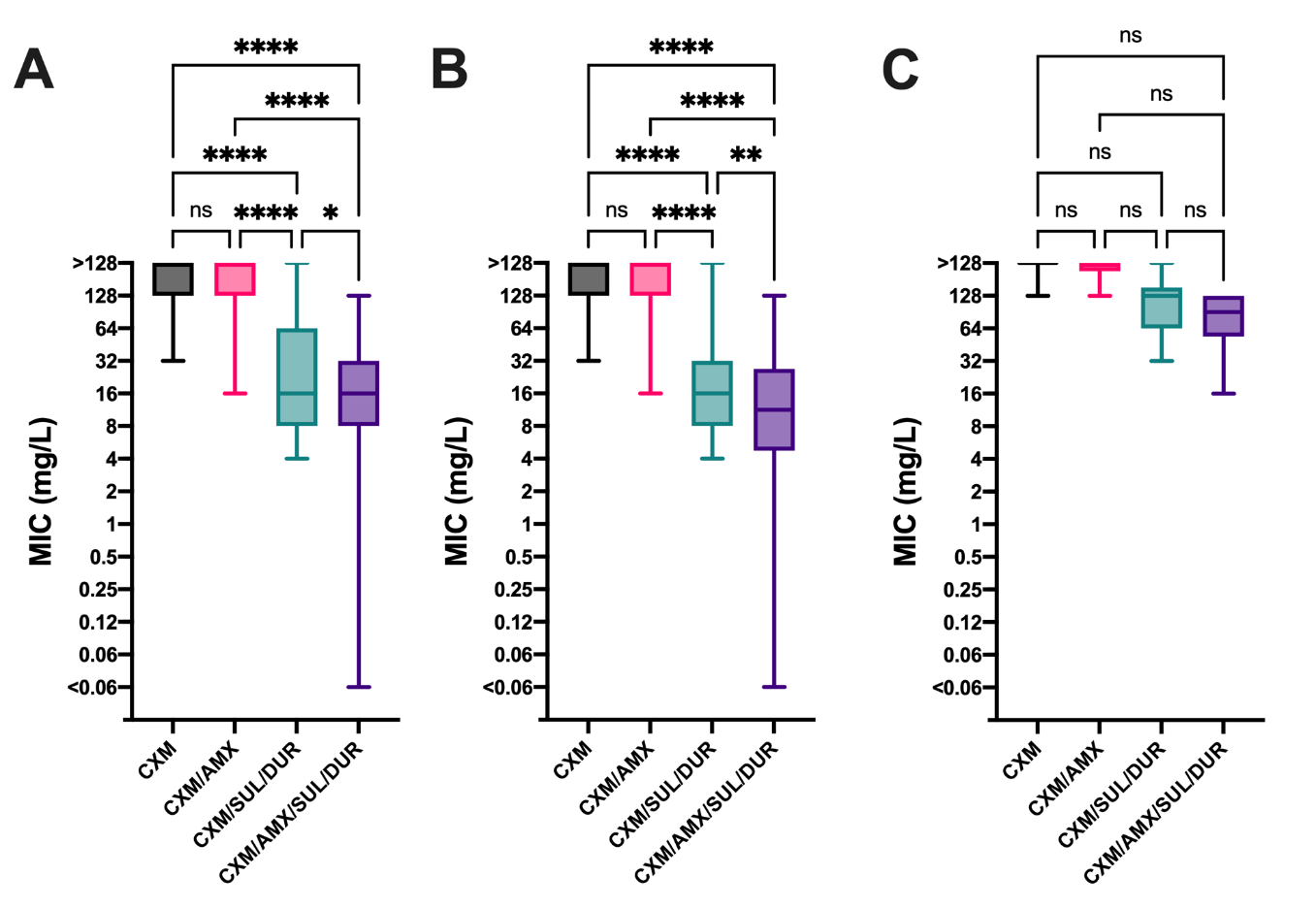


**Figure S1. Susceptibility of *M. abscessus* isolates to cefuroxime, cefuroxime/amoxicillin with and without sulbactam/durlobactam**. **A.** All isolates (n=50). **B.** Smooth isolates (n=40). **C.** Rough isolates (n=10). Amoxicillin added at a fixed concentration of 8 mg/L. Sulbactam/durlobactam added at a fixed concentration of 4/4 mg/L. CXM = cefuroxime, AMX = amoxicillin, SUL/DUR = sulbactam/durlobactam. ns= >0.05, * = p ≤ 0.05, ** = p ≤ 0.01, *** = p ≤ 0.001, **** = p ≤ 0.0001.


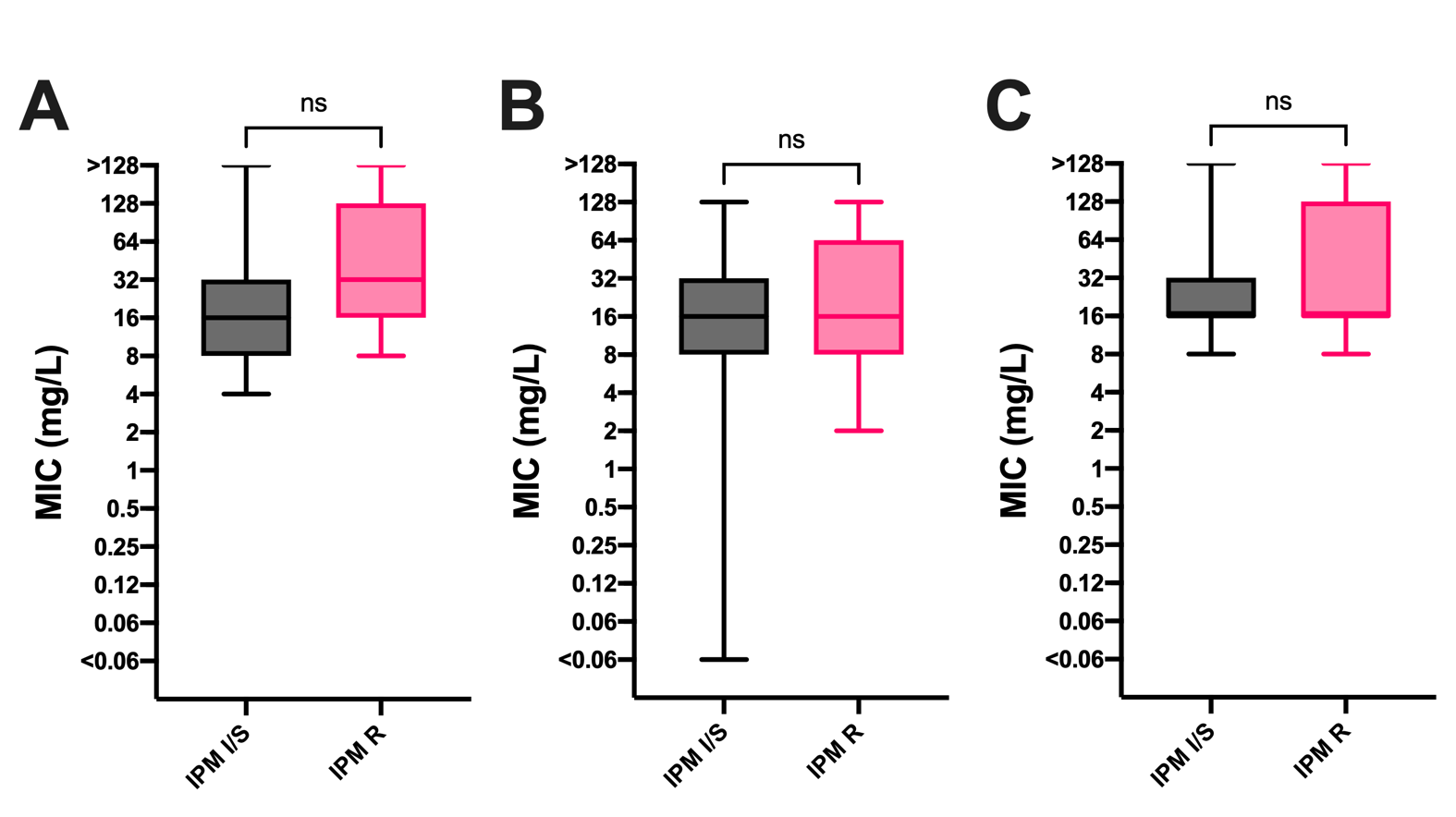


**Figure S2: Imipenem resistant isolates were not more resistant to sulbactam/durlobactam β-lactam combinations than other isolates. A.** Cefuroxime/ sulbactam-durlobactam. **B.** Cefuroxime/amoxicillin/sulbactam/durlobactam. **C.** Meropenem/sulbactam/durlobactam. IPM I/S = imipenem intermediate and susceptible isolates (n=39), IPM R = imipenem resistant isolates (n=11). Isolate imipenem susceptibility interpreted according to CLSI standards.^32^ ns = p>0.05.

##
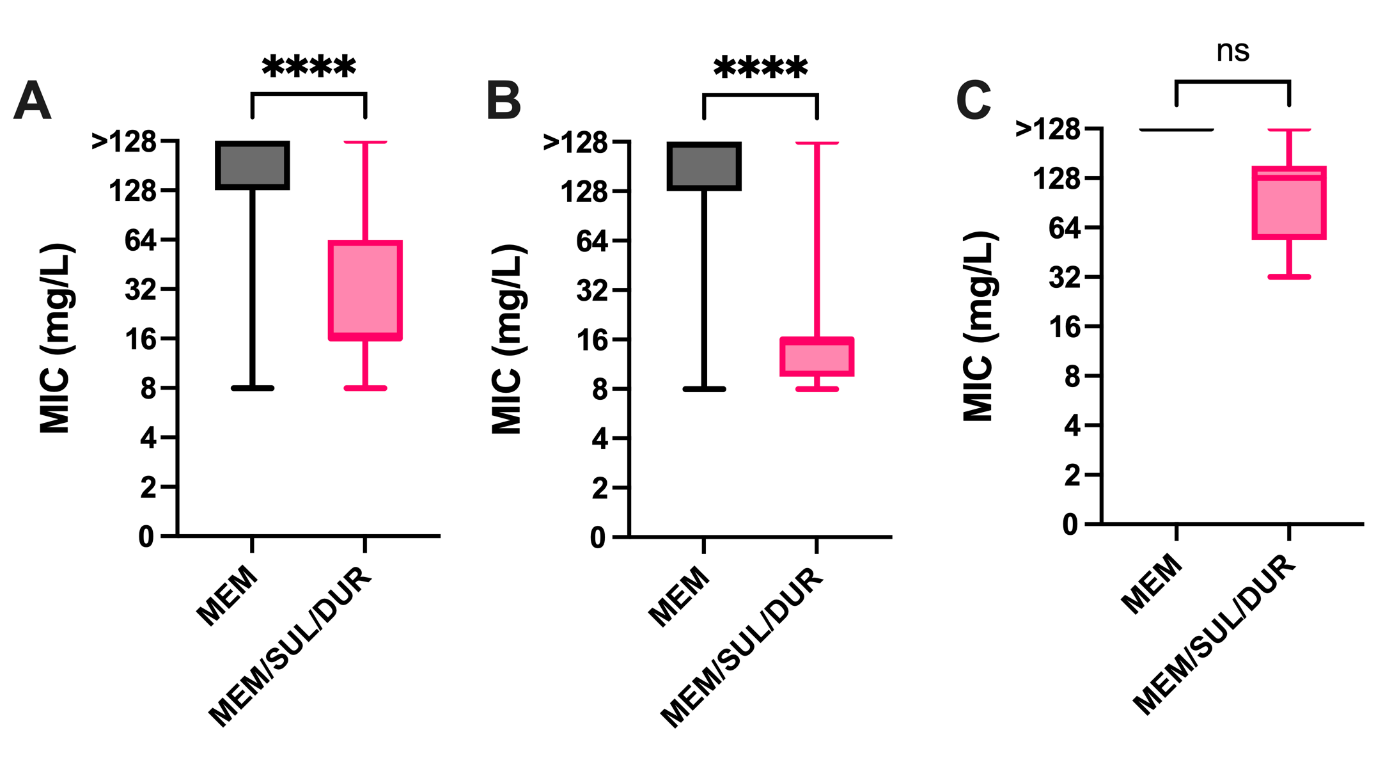


**Figure S3. Susceptibility of *M. abscessus* isolates to meropenem with and withoutsulbactam/durlobactam. A.** All isolates (n=50). **B.** Smooth isolates (n=40) . **C.** Rough isolates (n=10). Sulbactam/durlobactam added at a fixed concentration of 4/4 mg/L. MEM = meropenem, SUL/DUR = sulbactam/durlobactam. ns= >0.05, * = p ≤ 0.05, ** = p ≤ 0.01, *** = p ≤ 0.001, **** = p ≤ 0.0001.


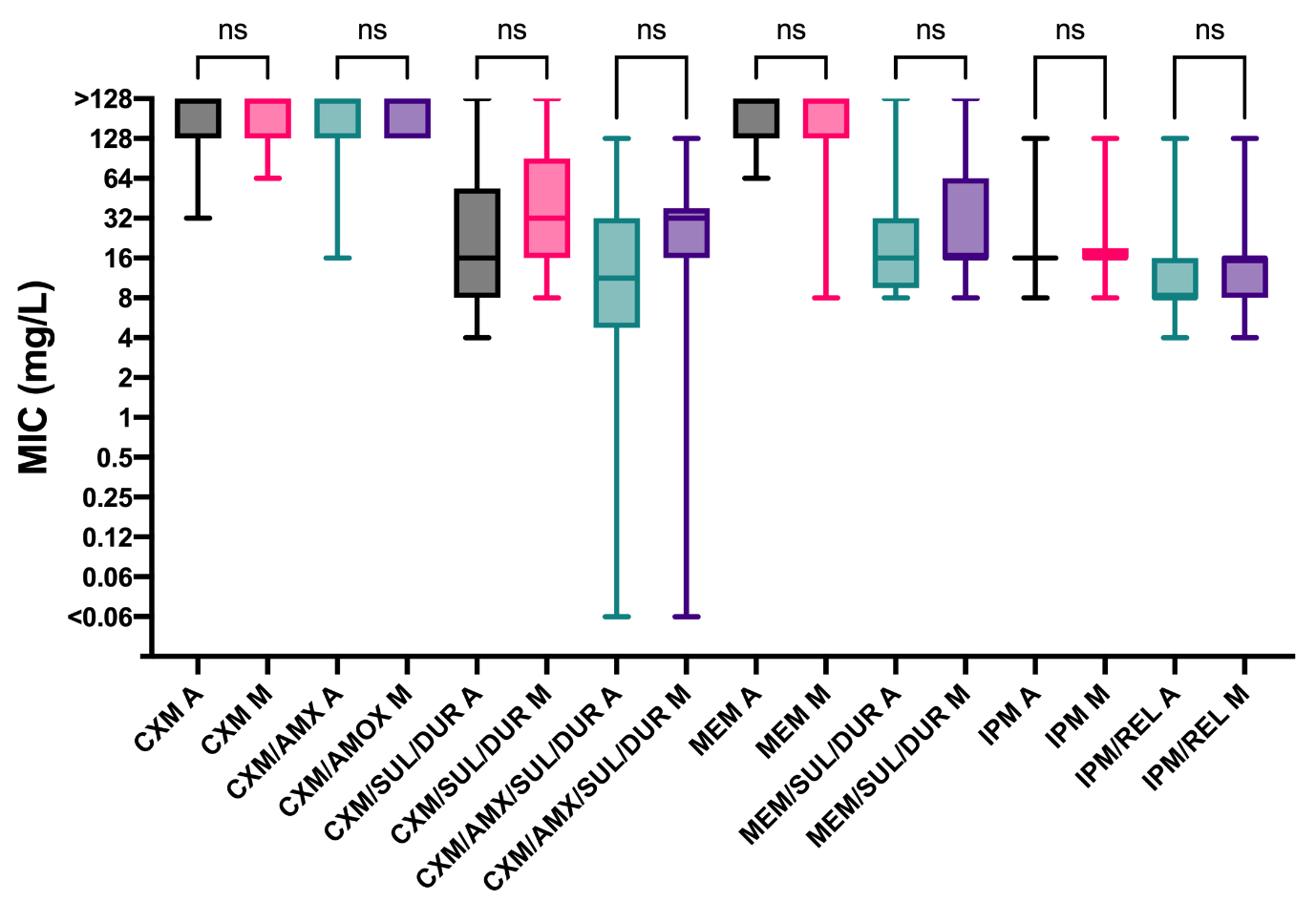


**Figure S4: There was no significant difference insusceptibility to the β-lactams tested between *M. abscessus* subspecies.** A = subsp. *abscessus* (n=36). M = subsp. *massiliense* (n=14). AMX = amoxicillin, CXM = cefuroxime, MEM = meropenem, SUL/DUR = sulbactam/durlobactam. Subspecies was determined by whole genome sequencing. ns= p >0.05


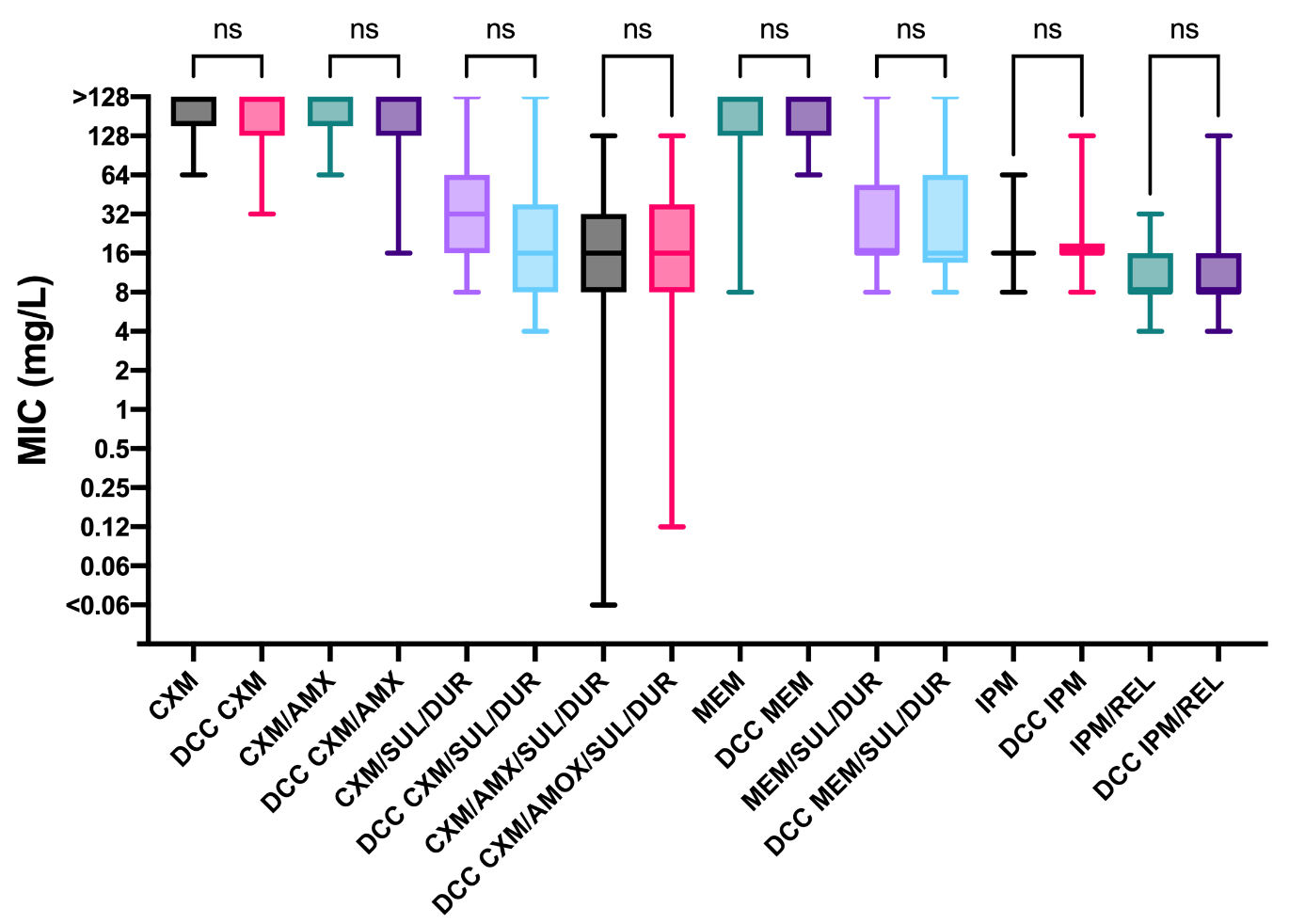


**Figure S5: Isolates assigned to a dominant circulating clone (DCC) did not have higher MICs for any of the β-lactams or sulbactam/durlobactam β-lactam combinations than other isolates.** DCC = isolates assigned to a dominant circulating clone after whole genome sequencing (n=34). AMX = amoxicillin, CXM = cefuroxime, MEM = meropenem, SUL/DUR = sulbactam/durlobactam. ns= p >0.05


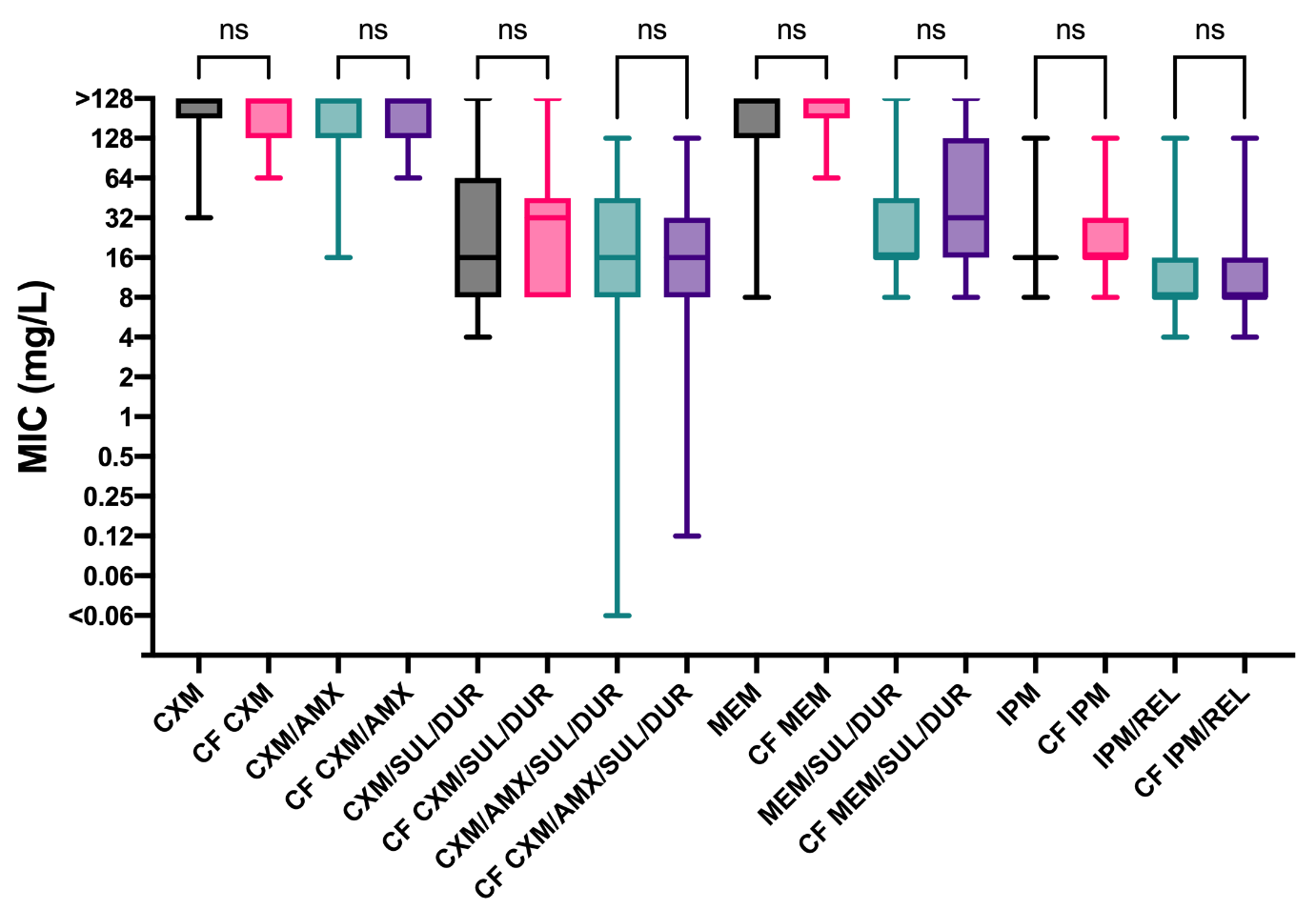


**Figure S6: Isolates from people with cystic fibrosis did not have higher MICs for any of the β-lactams or sulbactam/durlobactam β-lactam combinations than other isolates.** CF = susceptibility of isolates from patients with cystic fibrosis (n =25). AMX = amoxicillin, CXM = cefuroxime, MEM = meropenem, SUL/DUR = sulbactam/durlobactam. ns= p >0.05


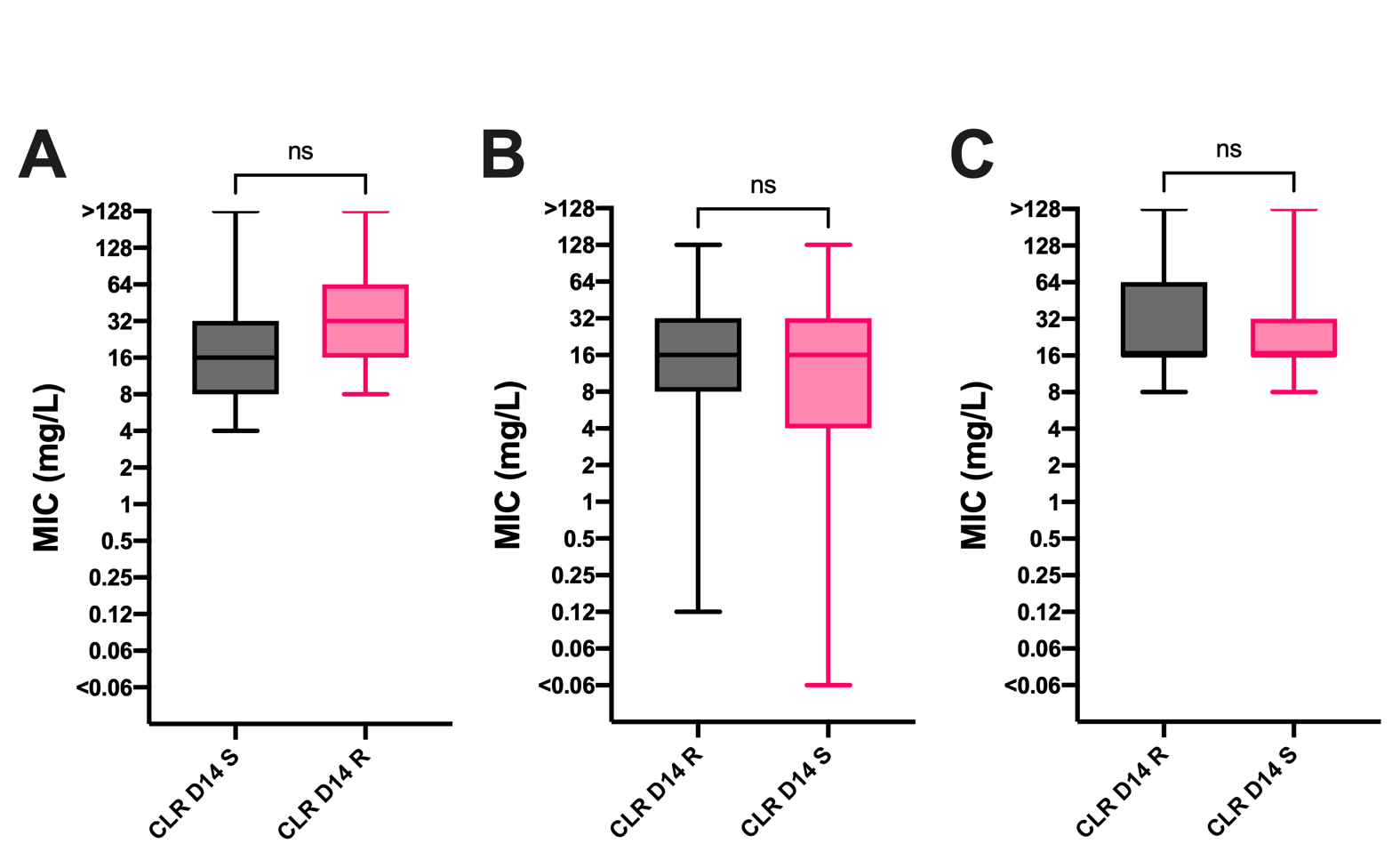


**Figure S7: Isolates with inducible clarithromycin resistance were not more resistant to sulbactam/durlobactam β-lactam combinations than other isolates. A.** Cefuroxime/sulbactam/durlobactam. **B.** Cefuroxime/amoxicillin/sulbactam/durlobactam. **C.** Meropenem/sulbactam/durlobactam. CLR D14 R = isolates with inducible clarithromycin resistance (n=27). CLR D14 S = isolates that were susceptible to clarithromycin (n=23). Isolate clarithromycin susceptibility interpreted according to CLSI standards.^32^ ns = p>0.05.


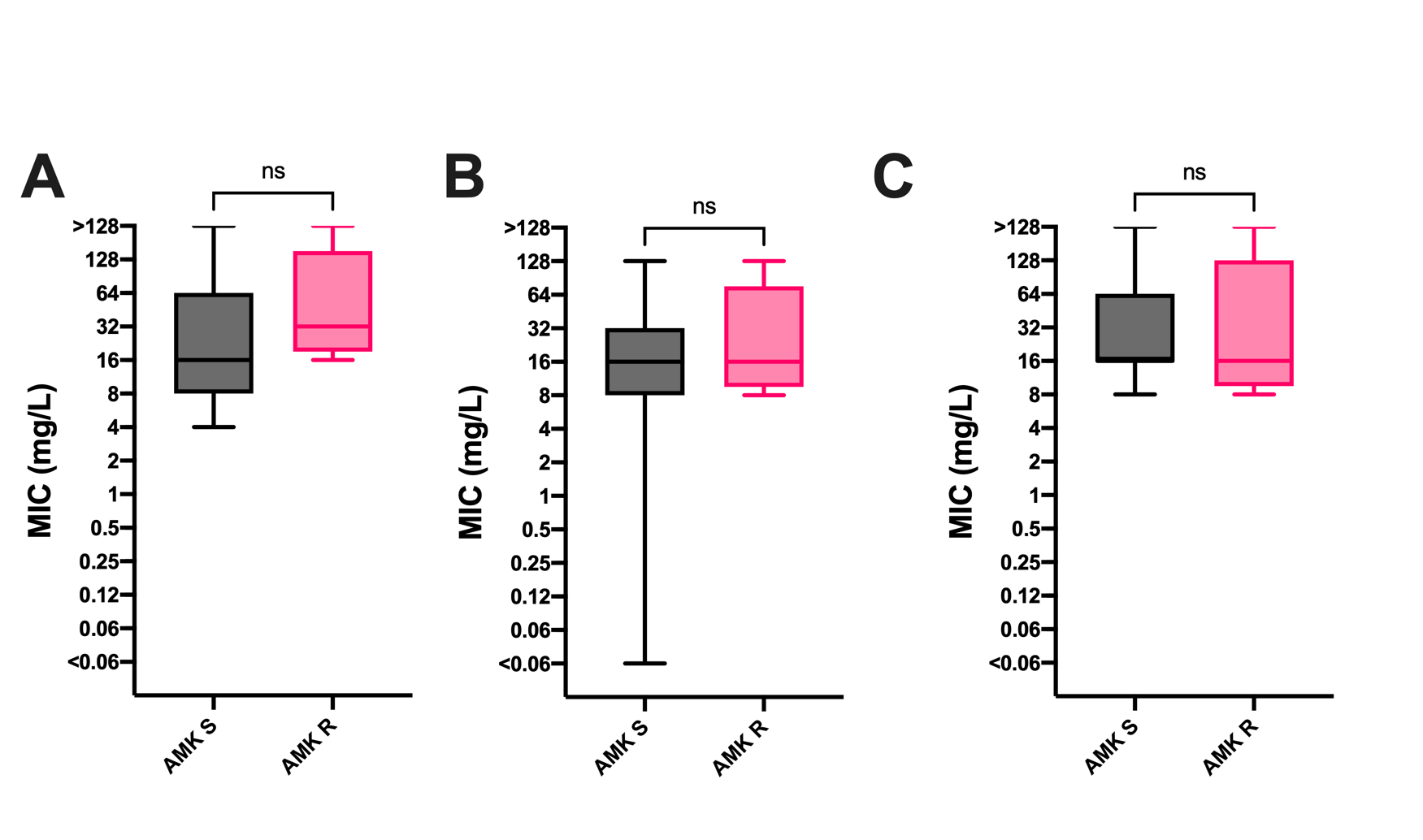


**Figure S8: Amikacin resistant isolates were not more resistant to sulbactam/durlobactam β-lactam combinations than other isolates. A.** Cefuroxime/sulbactam/durlobactam. **B.** Cefuroxime/amoxicillin/sulbactam/durlobactam. **C.** Meropenem/sulbactam/durlobactam. AMK S = amikacin susceptible isolates (n=46). AMK R = amikacin resistant isolates (n=4). Isolate amikacin susceptibility interpreted according to CLSI standards.^32^ ns = p>0.05.
